# Interleukin-6 and C-reactive protein, successful aging, and mortality: the PolSenior study

**DOI:** 10.1186/s12979-016-0076-x

**Published:** 2016-06-03

**Authors:** Monika Puzianowska-Kuźnicka, Magdalena Owczarz, Katarzyna Wieczorowska-Tobis, Pawel Nadrowski, Jerzy Chudek, Przemyslaw Slusarczyk, Anna Skalska, Marta Jonas, Edward Franek, Malgorzata Mossakowska

**Affiliations:** Department of Human Epigenetics, Mossakowski Medical Research Centre PAS, 5 Pawinskiego Street, 02-106 Warsaw, Poland; Department of Geriatrics and Gerontology, Medical Centre of Postgraduate Education, 01-826 Warsaw, Poland; PolSenior Project, International Institute of Molecular and Cell Biology, 02-109 Warsaw, Poland; Department of Palliative Medicine, Poznan University of Medical Sciences, 61-245 Poznan, Poland; Third Department of Cardiology, Medical University of Silesia in Katowice, 40-635 Katowice, Poland; Department of Pathophysiology, Faculty of Medicine, Medical University of Silesia in Katowice, 40-752 Katowice, Poland; Deparment of Internal Medicine and Oncological Chemotherapy, Faculty of Medicine, Medical University of Silesia in Katowice, 40-027 Katowice, Poland; Department of Internal Medicine and Geriatrics, Jagiellonian University Medical College, 31-351 Cracow, Poland

**Keywords:** Aging, Successful aging, Low-grade inflammation, Inflammaging, Interleukin 6 (IL-6), High sensitivity C-reactive protein (CRP), The Mini Mental State Examination (MMSE), The Katz Activity of Daily Living (ADL), Mortality

## Abstract

**Background:**

In the elderly, chronic low-grade inflammation (inflammaging) is a risk factor for the development of aging-related diseases and frailty. Using data from several thousand Eastern Europeans aged 65 years and older, we investigated whether the serum levels of two proinflammatory factors, interleukin-6 (IL-6) and C-reactive protein (CRP), were associated with physical and cognitive performance, and could predict mortality in successfully aging elderly.

**Results:**

IL-6 and CRP levels systematically increased in an age-dependent manner in the entire study group (IL-6: *n* = 3496 individuals, *p* < 0.001 and CRP: *n* = 3632, *p* = 0.003), and in the subgroup of successfully aging individuals who had never been diagnosed with cardiovascular disease, myocardial infarction, stroke, type 2 diabetes, or cancer, and had a Mini Mental State Examination (MMSE) score ≥24 and a Katz Activities of Daily Living (ADL) score ≥5 (IL-6: *n* = 1258, *p* < 0.001 and CRP: *n* = 1312, *p* < 0.001). In the subgroup of individuals suffering from aging-related diseases/disability, only IL-6 increased with age (IL-6: *n* = 2238, p < 0.001 and CRP: *n* = 2320, *p* = 0.249). IL-6 and CRP levels were lower in successfully aging individuals than in the remaining study participants (both *p* < 0.001). Higher IL-6 and CRP levels were associated with poorer physical performance (lower ADL score) and poorer cognitive performance (lower MMSE score) (both *p* < 0.001). This association remained significant after adjusting for age, gender, BMI, lipids, estimated glomerular filtration rate, and smoking status. Longer survival was associated with lower concentrations of IL-6 and CRP not only in individuals with aging-related diseases/disability (HR = 1.063 per each pg/mL, 95 % CI: 1.052-1.074, *p* < 0.001 and HR = 1.020 per each mg/L, 95 % CI: 1.015-1.025, *p* < 0.001, respectively) but also in the successfully aging subgroup (HR = 1.163 per each pg/mL, 95 % CI: 1.128-1.199, *p* < 0.001 and HR = 1.074 per each mg/L, 95 % CI: 1.047-1.100, *p* < 0.001, respectively). These associations remained significant after adjusting for age, gender, BMI, lipids and smoking status. The Kaplan-Meier survival curves showed similar results (all *p* < 0.001).

**Conclusions:**

Both IL-6 and CRP levels were good predictors of physical and cognitive performance and the risk of mortality in both the entire elderly population and in successfully aging individuals.

## Background

Immunosenescence is an integral part of human aging that results in a decrease in the number of naive T and B lymphocytes, the accumulation of memory and effector T and B cells, the production of defective antibodies, an increase in the production of autoantibodies, and in chronic low-grade inflammation (inflammaging) [1, 2]. Among the probable triggers of inflammaging are chronic viral infections, an aging-related increase in adiposity, dietary habits and aging-related changes in the composition of the gut microbiota [3–5]. Among its most important features are slight elevations of the concentrations of proinflammatory cytokines, chemokines, and adipokines, such as interleukin-1ß (IL-1ß), interleukin-6 (IL-6), tumor necrosis factor-α (TNF-α), and monocyte chemoattractant protein-1 (chemokine (C-C motif) ligand 2, CCL2) [6–8]. Proinflammatory cytokines stimulate the synthesis of C-reactive protein (CRP) in the liver, the level of which has been shown to increase in elderly individuals [9]. While clinical signs and symptoms of inflammaging are minimal or absent, this condition contributes to various molecular pathologies, leading to vascular damage and insulin resistance [10–12] and, therefore, increases the risk of developing type 2 diabetes, cardiovascular disease, stroke, cancer, sarcopenia, neurodegeneration, and frailty [13–22]. Furthermore, low-grade inflammation predicts mortality in elderly individuals who are affected by various pathologies [15, 17, 23–25].

IL-6 and CRP are among the most commonly used indicators of inflammation. However, while some published data suggest their causal or predictive roles in morbidity or mortality in the elderly, other studies do not support such views [26–29]. Moreover, the literature on the effects of inflammaging on mortality in successfully aging individuals is not extensive. Therefore, the main objective of the present work was to investigate whether the results of a single measurement of IL-6 and CRP levels were associated with mortality in elderly participants of the PolSenior study who were followed for an average of 4.3 years, with a particular emphasis on individuals who had never been diagnosed with aging-related diseases and remained in good physical and cognitive health. Another aim of this study was to determine possible associations between the concentrations of these factors and cognitive and physical functioning, which were assessed at the same time. Finally, we evaluated whether low-dose acetylsalicylic acid, which is frequently prescribed to seniors for the prevention of cardiovascular disease, affects IL-6 and CRP concentrations.

## Methods

### Material

The study group consisted of ≥65 year old seniors (*n* = 4979 individuals) who participated in the PolSenior study. The participants were randomly recruited in bundles in a stratified, proportional draw. The response rate was 42.6 %. The recruitment details are described elsewhere [30]. The respondents belonged to similarly-sized age groups (65–69, 70–74, 75–79, 80–84, 85–89 and ≥90 years old), each containing a similar number of males and females. A detailed questionnaire was used to obtain medical histories, current health status, socioeconomic status, demographic status, and lifestyle habits for all of the study participants. The participants also underwent a detailed examination, including elements of comprehensive geriatric assessment [30]. Individuals who had never been diagnosed with cardiovascular disease, myocardial infarction, stroke, type 2 diabetes, or cancer, and had a Mini Mental State Examination (MMSE) score ≥24 and Katz Activities of Daily Living (ADL) score ≥5 were classified as successfully aging. Of the entire group, blood samples were provided by 4101 individuals. Blood collection, surveys, and physical examinations were performed either on the same day or within a few days of each other. The survival of the study subjects was taken from the Population Register. This database was accessed in 2015, indicating that 1734 PolSenior participants (1318 of those who donated blood) had died. The total number of person-years of observation was 14,535.

The PolSenior project was approved by the Bioethics Commission of the Medical University of Silesia in Katowice. Each participant gave written, informed consent to participate in the study.

### Blood analysis

Venous blood was collected using a vacuum system and delivered in a cooler to local laboratories within 2 h, where serum and plasma samples were separated and frozen. All samples were then delivered to the Department of Human Epigenetics, Mossakowski Medical Research Centre in Warsaw. All of the IL-6 and CRP measurements were performed in the same laboratory. Interleukin-6 levels were measured in serum using ELISA (R&D System, Minneapolis, MN, USA, sensitivity 0.04 pg/mL) in 3895 individuals and CRP (high sensitivity CRP) levels were measured using a high-sensitivity immunoturbidymetric method (Modular PPE, Roche Diagnostics GmBH, Mannheim, Germany, sensitivity 0.11 mg/L) in 4093 individuals. In the other PolSenior participants, these measurements were not performed mostly because of refusal to provide blood or because an insufficient amount of serum was collected. Other biochemical parameters were assessed using routine techniques [30]. Study subjects with leukocyte counts >10,000/μL (indicating a considerable risk of ongoing infection) and/or who were being treated with glucocorticoids were excluded from further analyses. Therefore, analyses of IL-6 were performed in 3496 subjects, and of CRP were performed in 3632 subjects. In some of the study subjects, only IL-6 or only CRP was measured. The results were presented as median [1^st^ quartile, 3^rd^ quartile] values.

### Mini mental state examination and activities of daily living

Cognitive function was assessed using the MMSE [31] which was translated into Polish. The study participants were divided into five groups: normal cognition (MMSE score 28–30), minimal cognitive impairment (MMSE score 24–27), mild (MMSE score 20–23), moderate (MMSE score 10–19), or severe (MMSE score <10) cognitive impairment [32].

Physical performance was assessed using the ADL scale [33]. Based on the six domains of the ADL (transferring, feeding, bathing, dressing, personal hygiene and grooming, toileting), the study participants were divided into independent (ADL score 5–6), partially dependent (ADL score 3–4) and totally dependent (ADL score 0–2) [34].

### Statistical analysis

The statistical analyses were performed using Statistica 10 software (Statsoft Inc., Tulsa, OK, USA) and R software (R Foundation for Statistical Computing, Vienna, Austria) programs. Because of the skewed distribution of the IL-6 and CRP values, the variables were presented as median [1^st^ quartile, 3^rd^ quartile] values. Before the analyses, the IL-6 and CRP values were log-transformed. The significance of associations between the analyzed factors was tested using analysis of variance (ANOVA), and analysis of co-variance (ANCOVA) for multifactorial analyses. Pearson’s r was used as a measure of correlations between IL-6 and CRP levels.

A Kaplan-Meier plots were used to present survival curves, which were compared using the log-rank test. The Cox proportional hazards model was used for univariate survival analyses. In the multivariate survival analyses, age and body mass index (BMI) did not meet the assumption of proportional hazards. Therefore, the Cox proportional hazards model with time-dependent covariates was used. The hazard ratio (HR) of death and the 95 % confidence interval (CI) were calculated for associations between the analyzed variables and survival time.

For all of the statistical analyses, the level of significance was established at 0.05.

## Results

### Aging-associated changes in serum IL-6 and CRP levels

The mean age of all the study participants who met the inclusion criteria (3750 individuals, 47.8 % women, 52.2 % men) was 78.9 ± 8.6 years. The mean age of the successfully aging subgroup (35.4 % of the study population; 47.4 % women, 52.6 % men) was 76.3 ± 7.9 years, while in the remaining study participants who did not meet our definition of successful aging (64.6 % of the study population; 48 % women, 52 % men) was 80.3 ± 8.6 years. The age difference between the successfully aging participants and the remaining study participants was significant (*p* < 0.001).

Low-dose acetylsalicylic acid is commonly prescribed to seniors for the prevention of cardiovascular disease. We initially determined whether this medication affects IL-6 or CRP concentrations and whether individuals who received it should be included in the analysis or analyzed separately. The comparison between individuals who were not currently treated with low-dose acetylsalicylic acid *vs*. individuals who took this medication showed that the median concentrations of IL-6 were similar in both groups (2321 individuals, 2.3 pg/mL [1.5, 3.8] *vs*. 1175 individuals, 2.4 pg/mL [1.6, 3.8], respectively, *p* = 0.22), as were the median CRP values (2410 individuals, 2.3 mg/L [1.1, 4.8] *vs*. 1222 individuals, 2.3 mg/L [1.1, 4.7], respectively, *p* = 0.73). Therefore, all of the subsequent analyses were performed without considering whether the participants were treated with low-dose acetylsalicylic acid.

The median concentration of IL-6 in the entire study group (3496 individuals) was 2.3 pg/mL [1.5, 3.8]. In successfully aging study participants (1258 individuals) it was 2.0 pg/mL [1.3, 3.3], while in participants who suffered from aging-related diseases/disability (2238 individuals) it was 2.5 pg/mL [1.6, 4.2]. This difference was significant (*p* < 0.001). We also found that the older the age group, the higher was the median concentration of IL-6 not only in the entire study group and in individuals with aging-related diseases/disability, but also in the subgroup of successfully aging individuals (all *p* < 0.001, Table 1).

**Table 1:** IL-6 and CRP concentrations in ≥65 year-old seniors

Age (years)	All^a^		Successfully aging^b^		Others^c^	
	*n*	Concentration^d^	*n*	Concentration^d^	*n*	Concentration^d^
	IL-6 [pg/mL]					
65–69	588	1.8 [1.2, 2.9]	296	1.8 [1.1, 3.0]	292	1.9 [1.3, 2.9]
70–74	681	1.9 [1.3, 3.1]	308	1.7 [1.1, 2.8]	373	2.2 [1.4, 3.3]
75–79	596	2.1 [1.4, 3.4]	231	1.9 [1.3, 2.8]	365	2.4 [1.6, 3.6]
80–84	557	2.5 [1.7, 3.7]	163	2.4 [1.5, 3.5]	394	2.5 [1.7, 3.8]
85–89	598	2.8 [1.8, 4.9]	173	2.7 [1.8, 4.3]	425	2.8 [1.8, 5.1]
90+	476	3.5 [2.1, 5.5]	87	3.3 [2.3, 5.1]	389	3.5 [2.1, 5.6]
	CRP [mg/L]					
65–69	614	2.2 [1.1, 4.6]	311	2.0 [1.0, 4.1]	303	2.6 [1.2, 5.5]
70–74	706	2.1 [1.1, 4.1]	321	1.9 [1.0, 3.9]	385	2.2 [1.1, 4.4]
75–79	627	2.2 [1.1, 4.4]	245	1.9 [1.0, 3.8]	382	2.6 [1.2, 4.8]
80–84	572	2.4 [1.1, 5.2]	169	2.4 [1.0, 5.3]	403	2.3 [1.1, 5.2]
85–89	621	2.5 [1.1, 5.1]	177	2.8 [1.2, 4.6]	444	2.4 [1.1, 5.2]
90+	492	2.7 [1.1, 5.5]	89	2.6 [1.3, 5.3]	403	2.7 [1.1, 5.7]

^a^: Excluded are individuals with leukocytosis exceeding 10,000/μL or treated with glucocorticoids

^b^: Only individuals without past or current cancer, type 2 diabetes, cardiovascular disease/myocardial infarction/stroke, and with the MMSE score ≥24 and the ADL score 5–6

^c^: Individuals who did not meet the definition of successful aging

^d^: Concentrations of IL–6 and CRP are shown as median [1^st^ quartile, 3^rd^ quartile]

*n* number of individuals

The median CRP concentration in the entire study group (3632 individuals) was 2.3 mg/L [1.1, 4.7]. The subgroups of successfully aging participants (1312 individuals) and those who did not meet the definition of successful aging (2320 individuals) had median concentrations 2.1 mg/L [1.0, 4.3] and 2.5 mg/L [1.1, 5.1], respectively. This difference that was significant (*p* < 0.001). In the entire study group, we observed a significant increase in the median CRP concentration as age increased (*p* = 0.003). Notably, the median CRP concentration was higher in ≥80 year old study participants than in younger age groups. In the study participants with aging-related diseases/disability, the median CRP level was not associated with age (*p* = 0.25). In the successfully aging subgroup, the median CRP levels were also higher in the older age groups, but the level of significance has not been reached (*p* = 0.09) (Table 1).

IL-6 and CRP levels were correlated with each other in the entire study group (r = 0.502, *p* < 0.001), the successfully aging subgroup (*r* = 0.538, *p* < 0.001), and study participants with aging-related diseases/disability (*r* = 0.504, *p* < 0.001).

We also evaluated whether IL-6 and CRP concentrations differed in women and men and found that the median IL-6 concentrations were higher in men (1673 women, 2.2 pg/mL [1.5, 3.6] *vs*. 1823 men, 2.4 pg/mL [1.6, 4.0], *p* = 0.006), whereas the median CRP concentrations were not significantly different (1735 women, 2.4 mg/L [1.2, 4.7] *vs*. 1897 men, 2.2 mg/L [1.0, 4.9], *p* = 0.25).

### Association between the IL-6 and CRP levels and other biochemical parameters and functional and cognitive performance

IL-6 concentrations were significantly associated with BMI (*p* < 0.001 for the entire study group, *p* = 0.015 for the successfully aging subgroup, and *p* = 0.007 for study participants with aging-related diseases/disability). Notably, in individuals with a BMI <18.5 kg/m^2^, the median IL-6 value was the highest, while in overweight individuals it was the lowest, and again increased in the obese. In addition, the median IL-6 value was associated with estimated glomerular filtration rate (eGFR) (*p* < 0.001 for the entire study group, *p* < 0.001 for the successfully aging subgroup, and *p* < 0.001 for study participants with aging-related diseases/disability) and was the highest in individuals with the lowest eGFR, as well as with heart rate (*p* < 0.001, *p* < 0.001 and *p* < 0.001) where it was the highest in individuals with a heart rate >80/min. It was also associated with smoking status (*p* = 0.02 for the entire study group and *p* = 0.016 for participants with aging-related diseases/disability); it was the highest in current smokers. No association with smoking status was found in the successfully aging subgroup. Of note, the IL-6 concentration was negatively associated with total cholesterol levels (*p* < 0.001 for the entire study group, *p* < 0.001 for the successfully aging subgroup, and *p* < 0.001 for study participants with aging-related diseases/disability), LDL cholesterol (*p* < 0.001, *p* = 0.007 and *p* < 0.001), and HDL cholesterol (*p* < 0.001, *p* < 0.001 and *p* < 0.001) (Table 2). The multifactorial analysis including age, gender, BMI, HDL, LDL, eGFR and smoking status, showed that only gender lost its significant association with IL-6 in the entire study group. In the successfully aging subgroup, gender and LDL lost their associations with IL-6, while in participants with aging-related diseases/disability the association was lost for gender and eGFR. The other factors remained significantly associated with IL-6.

**Table 2:** IL-6 and CRP concentration in ≥65 year-old seniors in relation to select clinical and biochemical parameters, and functional status

		IL-6 [pg/mL]						CRP [mg/L]					
		All^a^		Successfully aging^b^		Others^c^		All^a^		Successfully aging^b^		Others^c^	
		n	Concentration^d^	n	Concentration^d^	n	Concentration^d^	n	Concentration^d^	n	Concentration^d^	n	Concentration^d^
BMI [kg/m^2^]	<18.5	60	3.2 [1.8, 5.4]	21	3.2 [1.7, 4.8]	39	3.2 [1.9, 5.7]	64	2.3 [1.0, 7.4]	21	1.8 [0.7, 7.5]	43	2.5 [1.0, 7.2]
	18.5-24.9	814	2.3 [1.4, 3.9]	315	1.9 [1.2, 3.2]	499	2.5 [1.6, 4.5]	865	1.7 [0.8, 3.8]	336	1.5 [0.8, 3.0]	529	1.8 [0.9, 4.3]
	25–29.9	1381	2.2 [1.4, 3.5]	548	1.9 [1.3, 3.2]	833	2.3 [1.5, 3.8]	1425	2.1 [1.0, 4.2]	569	2.0 [1.0, 4.0]	856	2.2 [1.0, 4.3]
	30–39.9	997	2.3 [1.6, 3.6]	343	2.2 [1.4, 3.6]	654	2.4 [1.7, 3.6]	1025	2.8 [1.5, 5.2]	354	2.9 [1.6, 5.4]	671	2.7 [1.4, 5.1]
	≥40	69	2.8 [2.1, 4.1]	17	2.5 [1.9, 3.4]	52	2.9 [2.1, 4.9]	69	4.2 [2.2, 8.8]	17	4.3 [2.5, 7.8]	52	4.1 [2.1, 9.1]
Cholesterol [mg/dL]	<175	1029	2.7 [1.7, 4.8]	256	2.4 [1.5, 4.4]	773	2.8 [1.8, 5.1]	1085	2.3 [1.0, 5.3]	267	2.2 [1.0, 5.0]	818	2.4 [1.0, 5.3]
	175–189	452	2.3 [1.6, 3.8]	178	2.2 [1.4, 3.7]	274	2.4 [1.7, 3.8]	478	2.4 [1.1, 5.1]	184	1.9 [1.0, 4.2]	294	2.6 [1.1, 5.7]
	190–239	1302	2.2 [1.5, 3.5]	512	2.0 [1.3, 3.1]	790	2.4 [1.6, 3.8]	1375	2.2 [1.1, 4.4]	543	2.2 [1.0, 4.1]	832	2.3 [1.1, 4.7]
	240–309	593	2.0 [1.4, 3.1]	275	1.8 [1.2, 2.7]	318	2.2 [1.5, 3.4]	627	2.3 [1.2, 4.5]	291	2.1 [1.2, 4.0]	336	2.7 [1.4, 5.2]
	≥310	65	2.2 [1.4, 3.6]	27	2.4 [1.4, 3.7]	38	2.2 [1.5, 3.6]	67	3.3 [1.5, 5.2]	27	2.1 [1.0, 5.0]	40	3.6 [1.7, 5.3]
HDL [mg/dL]	<40	774	3.0 [1.8, 4.9]	199	2.7 [1.6, 3.9]	575	3.1 [1.9, 5.3]	822	2.9 [1.4, 6.2]	211	2.6 [1.3, 5.4]	611	3.0 [1.5, 6.5]
	40–44	525	2.6 [1.7, 4.4]	168	2.4 [1.6, 4.0]	357	2.7 [1.8, 4.5]	549	2.4 [1.2, 5.0]	176	2.3 [1.3, 4.7]	373	2.4 [1.2, 5.1]
	45–49	528	2.4 [1.6, 4.0]	190	2.1 [1.3, 3.5]	338	2.6 [1.8, 4.2]	549	2.5 [1.1, 5.3]	201	2.5 [1.1, 4.8]	348	2.4 [1.2, 5.9]
	50–59	851	2.1 [1.4, 3.4]	362	1.9 [1.3, 3.2]	489	2.2 [1.5, 3.4]	900	2.2 [1.1, 4.2]	370	2.1 [1.0, 3.9]	530	2.3 [1.1, 4.3]
	≥60	760	1.8 [1.2, 3.0]	328	1.7 [1.1, 2.6]	432	2.0 [1.3, 3.3]	810	1.8 [0.8, 3.6]	353	1.6 [0.8, 3.1]	457	1.9 [0.9, 4.1]
LDL [mg/dL]	<70	329	2.8 [1.8, 5.1]	53	2.2 [1.6, 3.6]	276	2.9 [1.9, 5.5]	347	2.4 [0.9, 4.9]	55	2.4 [0.7, 5.0]	292	2.4 [1.0, 4.9]
	71–99	809	2.5 [1.6, 4.2]	234	2.2 [1.5, 4.1]	575	2.6 [1.6, 4.2]	860	2.3 [1.0, 4.7]	249	1.9 [1.0, 4.3]	611	2.4 [1.0, 5.0]
	100–114	506	2.3 [1.6, 4.0]	204	2.1 [1.3, 3.6]	302	2.5 [1.7, 4.3]	530	2.1 [1.0, 4.8]	214	2.1 [1.0, 4.2]	316	2.1 [1.0, 5.1]
	115–154	1168	2.2 [1.4, 3.5]	463	1.9 [1.3, 3.1]	705	2.4 [1.6, 3.7]	1234	2.2 [1.1, 4.6]	485	2.1 [1.0, 4.0]	749	2.5 [1.2, 5.1]
	155–189	468	2.1 [1.5, 3.3]	218	1.9 [1.3, 2.9]	250	2.2 [1.6, 3.8]	494	2.5 [1.4, 4.6]	230	2.3 [1.2, 4.1]	264	2.7 [1.5, 5.5]
	≥190	159	2.1 [1.3, 3.2]	75	1.8 [1.2, 3.0]	84	2.2 [1.5, 3.4]	166	2.7 [1.2, 5.4]	78	2.3 [1.2, 5.4]	88	3.0 [1.3, 5.4]
Glucose [mg/dL]	<100	2133	2.3 [1.5, 3.8]	924	2.0 [1.3, 3.3]	1209	2.5 [1.7, 4.4]	2261	2.2 [1.0, 4.6]	966	2.1 [1.0, 4.1]	1295	2.4 [1.1, 5.0]
	100–139.9	1042	2.4 [1.6, 3.7]	324	2.1 [1.4, 3.4]	718	2.5 [1.6, 3.8]	1089	2.3 [1.1, 4.8]	346	2.2 [1.1, 4.8]	743	2.4 [1.1, 4.9]
	≥140	256	2.5 [1.7, 4.1]	na	na	256	2.5 [1.7, 4.1]	272	3.0 [1.4, 6.8]	na	na	272	3.0 [1.4, 6.8]
eGFR [mL/min/ 1.73 m^2^]	<45	359	3.2 [2.1, 5.3]	70	3.8 [2.6, 6.7]	289	3.0 [2.0, 5.2]	378	3.2 [1.5, 6.3]	77	3.5 [1.9, 6.3]	301	3.0 [1.5, 6.3]
	45–60	639	2.7 [1.7, 4.1]	187	2.4 [1.5, 3.6]	452	2.8 [1.8, 4.5]	668	2.5 [1.1, 5.4]	192	2.2 [1.1, 4.4]	476	2.7 [1.2, 6.2]
	>60	2421	2.1 [1.4, 3.5]	977	1.9 [1.3, 3.1]	1444	2.3 [1.6, 3.8]	2531	2.2 [1.0, 4.4]	1019	2.0 [1.0, 4.1]	1512	2.3 [1.0, 4.7]
Heart rate [per min]	<60	341	2.2 [1.5, 3.6]	126	1.8 [1.2, 2.9]	215	2.6 [1.6, 4.0]	354	1.6 [0.9, 3.6]	131	1.8 [1.0, 3.6]	223	1.5 [0.9, 3.6]
	60–80	2374	2.2 [1.5, 3.6]	893	2.0 [1.3, 3.2]	1481	2.3 [1.6, 3.8]	2463	2.3 [1.0, 4.5]	929	2.1 [1.0, 4.0]	1534	2.4 [1.1, 4.9]
	>80	768	2.7 [1.7, 4.8]	239	2.4 [1.5, 4.0]	529	2.8 [1.8, 5.1]	801	3.0 [1.4, 6.2]	252	2.7 [1.3, 5.6]	549	3.1 [1.4, 6.3]
Smoking	Never	1968	2.3 [1.5, 3.7]	689	1.9 [1.3, 3.1]	1279	2.4 [1.6, 4.1]	2035	2.3 [1.1, 4.5]	710	2.1 [1.0, 3.9]	1325	2.4 [1.1, 4.9]
	Past	1196	2.4 [1.5, 3.8]	424	2.0 [1.4, 3.3]	772	2.5 [1.6, 4.0]	1250	2.2 [1.0, 4.8]	450	2.1 [1.0, 4.5]	800	2.3 [1.0, 5.0]
	Current	304	2.7 [1.7, 4.5]	140	2.4 [1.4, 3.7]	164	3.0 [1.8, 5.2]	319	3.1 [1.3, 6.2]	147	2.4 [1.1, 5.1]	172	3.4 [1.5, 7.7]
MMSE [points]	28–30	1121	1.9 [1.3, 3.1]	606	1.9 [1.2, 3.0]	515	2.0 [1.4, 3.1]	1153	2.0 [1.0, 4.1]	629	1.9 [1.0, 4.1]	524	2.1 [1.0, 4.1]
	24–27	1245	2.3 [1.5, 3.7]	637	2.2 [1.4, 3.6]	608	2.4 [1.6, 4.0]	1302	2.3 [1.1, 4.6]	668	2.3 [1.1, 4.4]	634	2.5 [1.1, 5.1]
	20–23	593	2.6 [1.7, 4.3]	na	na	593	2.6 [1.7, 4.3]	617	2.4 [1.2, 5.2]	na	na	617	2.4 [1.2, 5.2]
	10–19	324	3.0 [2.0, 5.5]	na	na	324	3.0 [2.0, 5.5]	348	2.5 [1.0, 5.4]	na	na	348	2.5 [1.0, 5.4]
	<10	111	3.3 [2.1, 6.0]	na	na	111	3.3 [2.1, 6.0]	114	3.7 [1.5, 8.3]	na	na	114	3.7 [1.5, 8.3]
ADL [points]	5–6	3112	2.2 [1.5, 3.6]	1258	2.0 [1.3, 3.3]	1854	2.3 [1.6, 3.7]	3220	2.2 [1.0, 4.4]	1312	2.1 [1.0, 4.3]	1908	2.2 [1.0, 4.5]
	3–4	205	3.2 [2.2, 6.2]	na	na	205	3.2 [2.2, 6.2]	220	3.4 [1.7, 9.3]	na	na	220	3.4 [1.7, 9.3]
	0–2	165	4.2 [2.6, 7.4]	na	na	165	4.2 [2.6, 7.4]	178	3.9 [1.9, 9.4]	na	na	178	3.9 [1.9, 9.4]

^a^: Excluded are individuals with leukocytosis exceeding 10,000/mm^3^ or treated with glucocorticoids

^b^: Only individuals without past or current cancer, type 2 diabetes, cardiovascular disease/myocardial infarction/stroke, and with the MMSE score ≥24 and the ADL score 5–6

^c^: Individuals who did not meet the definition of successful aging

^d^: Concentrations of IL–6 and CRP are shown as median [1^st^ quartile, 3^rd^ quartile]

*n* Number of individuals

*na* Not applicable

We also evaluated whether IL-6 concentrations were related to functional and cognitive performance. We found that higher IL-6 levels were associated with poorer physical performance (lower ADL score) and poorer cognitive performance (lower MMSE score) (all *p* < 0.001 for the entire study group as well as for both subgroups) (Table 2). He multifactorial analyses that were adjusted for age, gender, BMI, HDL, LDL, eGFR and smoking status revealed that ADL and MMSE scores in the entire studied group remained associated with IL-6 (*p* = 0.006 and *p* = 0.007, respectively). Such an analysis was not performed separately in the successfully aging subgroup because only individuals with the highest ADL and MMSE scores, reflecting good physical and cognitive performance, were included in this subgroup.

The CRP concentration was significantly associated with BMI (*p* < 0.001 for the entire study group, *p* < 0.001 for the successfully aging subgroup, and *p* < 0.001 for study participants with aging-related diseases/disability). The CRP concentration was the lowest in the group of normal-weight individuals and then systematically increased as BMI increased. Notably, it was also higher in individuals with a BMI <18.5 kg/m^2^ compared with normal-weight study subjects. Additionally, the CRP concentration was associated with fasting glucose in the entire study group (*p* = 0.003) and individuals with aging-related diseases/disability (*p* = 0.006) and was the highest in individuals with a glucose level ≥140 mg/dL. Notably, in the successfully aging subgroup no association was found. The CRP concentration was also associated with eGFR (*p* < 0.001 for the entire study group, *p* < 0.001 for the successfully aging subgroup, and *p* < 0.001 for participants with aging-related diseases/disability) and was the highest in individuals with the lowest eGFR, as well as with heart rate (*p* < 0.001, *p* < 0.001 and *p* < 0.001) where it was the highest in individuals with a heart rate >80/min. Furthermore, the CRP concentration was also associated with smoking status in the entire study group (*p* = 0.005) and in individuals with aging-related diseases/disability (*p* = 0.002) where it was the highest in current smokers; however, no association with smoking status was found in the successfully aging subgroup. Finally, The CRP concentration was negatively associated with the level of HDL cholesterol (*p* < 0.001, *p* < 0.001 and *p* < 0.001) (Table 2). The multifactorial analysis including age, gender, BMI, HDL, eGFR and smoking status revealed that all of the factors remained significantly associated with CRP in the entire study group and successfully aging subgroup, whereas age lost its significant association in the subgroup of participants with aging-related diseases/disability.

We also found that higher CRP levels were associated with poorer physical performance (lower ADL score) and poorer cognitive performance (lower MMSE score) (all *p* < 0.001 for the entire study group as well as for both subgroups, Table 2). The multifactorial analyses that were adjusted for age, gender, BMI, HDL, eGFR and smoking status revealed that in the entire studied group the ADL and MMSE scores remained related to CRP at statistically significant level (*p* = 0.019 and *p* < 0.001 respectively). We did not perform such an analysis in the successfully aging subgroup because only individuals with the highest ADL and MMSE scores were included in this subgroup.

### Association between IL-6 and CRP levels and survival

The 1-year mortality rate was 6.6 %, 2.6 %, and 8.8 % for the entire study group, the successfully aging subgroup, and study participants with aging-related diseases/disability, respectively. In the univariate analysis, longer survival was associated with a lower IL-6 concentration: HR = 1.077 per each pg/mL (95 % CI: 1.068–1.086, *p* < 0.001) in the entire study group, HR = 1.163 per each pg/mL (95 % CI: 1.128–1.199, *p* < 0.001) in the successfully aging subgroup, and HR = 1.063 per each pg/mL (95 % CI: 1.052–1.074, *p* < 0.001) in individuals with aging-related diseases/disability. Longer survival was also associated with a lower CRP concentration: HR = 1.025 per each mg/L (95 % CI: 1.020–1.029, *p* < 0.001) in the entire study group, HR = 1.074 per each mg/L (95 % CI: 1.047–1.100, *p* < 0.001) in successfully aging study participants, and HR = 1.020 per each pg/mL (95 % CI: 1.015–1.025, *p* < 0.001) in the remaining participants.

**Fig. 1:**
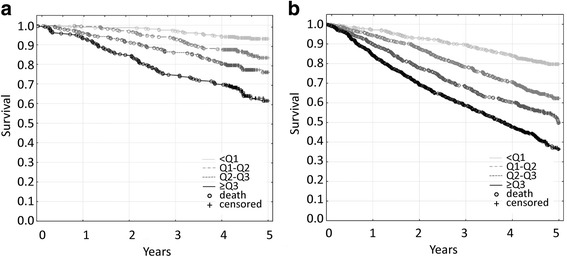
Kaplan-Meier survival curves for the elderly population that was classified according to quartiles of IL-6 concentrations. **a**. In the successfully aging subgroup, the 1-year mortality rates were 1.0 % for the <1^st^ quartile, 0.6 % for the 1^st^-2^nd^ quartile, 4.1 % for the 2^nd^-3^rd^ quartile, and 6.8 % for the >3^rd^ quartile. The differences between the <1^st^ quartile and all of the other quartiles were significant (all *p* < 0.001). **b**. In the subgroup of individuals who suffered from aging-related diseases/disability, the 1-year mortality rates were 2.9 % for the <1^st^ quartile, 5.7 % for the 1^st^-2^nd^ quartile, 10.0 % for the 2^nd^-3^rd^ quartile, and 15.8 % for the >3^rd^ quartile. The differences between the <1^st^ quartile and all of the other quartiles were significant (all *p* < 0.001)

**Fig. 2:**
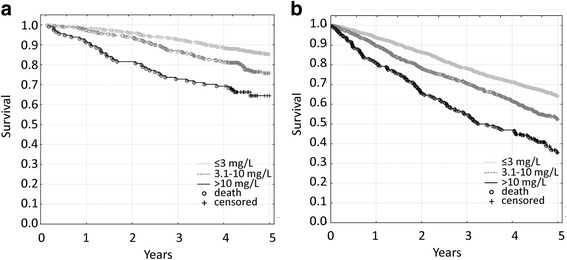
Kaplan-Meier survival curves for the elderly population that was classified according to CRP concentrations. **a**. In the successfully aging subgroup, the 1-year mortality rates were 1.6 % for ≤3 mg/L, 2.8 % for 3.1–10 mg/L, and 8.8 % for >10 mg/L. The differences between ≤3 mg/L and the other concentration ranges were significant (all *p* < 0.001). **b**. In the subgroup of individuals who suffered from aging-related diseases/disability, the 1-year mortality rates were 6.3 % for ≤3 mg/L, 10.3 % for 3.1–10 mg/L, and 19.2 % for >10 mg/L. The differences between ≤3 mg/L and other concentration ranges were significant (all *p* < 0.001)

The results of the univariate analysis were consistent with the Kaplan-Meier survival curves, in which higher concentrations of IL-6 and CRP were associated with a lower probability of survival in the entire group of seniors, in successfully aging ones, as well as in individuals with aging-related diseases/disability (all *p* < 0.001) (Figs. 1, 2).

A statistical model was applied that included IL-6 value, gender, age, BMI, HDL, LDL, eGFR and smoking status. We found that longer survival was associated with a lower IL-6 concentration, even after adjusting for these covariates, in the entire study group: HR = 1.042 per each pg/mL (95 % CI: 1.029–1.055, *p* < 0.001), the successfully aging subgroup: HR = 1.112 per each pg/mL (95 % CI: 1.069–1.155, *p* < 0.001), as well as in study participants with aging-related diseases/disability: HR = 1.031 per each pg/mL (95 % CI: 1.018–1.045, *p* < 0.001).

We also tested a model that included CRP value, gender, age, BMI, HDL, eGFR and smoking status, and found that longer survival was associated with a lower CRP concentration after adjusting for these factors in the entire group of seniors: HR = 1.017 per each mg/L (95 % CI: 1.012–1.023, *p* < 0.001), in the successfully aging subgroup: HR = 1.035 per each mg/L (95 % CI: 1.021–1.050, *p* < 0.001), and in the remaining study participants with aging-related diseases/disability: HR = 1.013 per each pg/mL (95 % CI: 1.007–1.019, *p* < 0.001).

## Discussion

It is well documented that chronic inflammation is associated with aging-related morbidity [13–22], and with mortality in affected individuals [15, 17, 23–25]. Therefore, the present study evaluated a large group of Eastern European Caucasian seniors to determine whether results of a single measurement of the levels of two proinflammatory factors, IL-6 and CRP, were associated with biochemical and functional parameters and whether they were predictors of mortality. We performed this analysis for the entire study group of ≥65 year-old seniors who were not preselected on the basis of their health and functional status, as well as for subgroups of successfully aging individuals and individuals with aging-related chronic diseases/disability.

There is no single generally agreed upon definition of successful aging. It is usually defined as survival to an older age while being free of aging-associated diseases, such as cardiovascular disease, cancer, neurodegeneration, and type 2 diabetes, with good physical and cognitive functioning [35]. This “biological” definition is sometimes augmented by a requirement to have good social functioning and high life satisfaction [35]. However, for the purpose of this study, in which we analyzed biological factors associated with aging, we used the most common definition that is limited to the biology of aging. An additional reason for such limitation was that social functioning and life satisfaction might be significantly influenced by non-biological factors such as income, family situation, or place of residence. We found that IL-6 and CRP levels were good predictors of physical and cognitive performance and mortality not only in the entire aging study group and in individuals with aging-related diseases/disability, but also in individuals who were aging successfully.

IL-6 is known to induce the production of CRP in the liver [36]. Therefore, as expected, the levels of IL-6 and CRP were correlated in the elderly subjects in this study. However, aging-related changes in CRP did not accurately reflect changes in IL-6. Impaired liver function, which could be expected mainly in the subgroup of individuals who did not age successfully, might affect CRP production. We tested this hypothesis by correlating aspartate transaminase (AST) and alanine transaminase (ALT) levels with the CRP level and found that some negative correlations were significant, but all of the correlation coefficients were extremely low (data not shown). Therefore, we conclude that liver function had no significant influence on CRP production in our study group. Another plausible explanation for the lack of complete correspondence between IL-6 and CRP concentrations is that CRP production is affected by other cytokines, such as IL-1 and IL-17 [36], which were not measured in the present study.

Notably, although statistically significant, the differences between mean IL-6 and CRP concentrations in the successfully aging participants and remaining study participants who suffered form from aging-related diseases/disability were only approximately 20 % (2 pg/mL *vs*. 2.5 pg/mL and 2.1 mg/L *vs*. 2.5 mg/L, respectively). Therefore, the question arises as to the reason why, despite such a small differences, some individuals experienced healthy aging, while others suffered from aging-related diseases. The most likely explanation for this phenomenon is that the phenotype of aging results not only from the amount of the examined proinflammatory factors, but also from a balance between numerous proinflammatory and antiinflammatory agents (inflammaging *vs*. anti-inflammaging) [37, 38]. Moreover, the effect of such a balance might be modified by other conditions, such as diet, body fat content, level of physical activity, smoking, and other disease risk factors, including genetic background [39–42].

Another important finding of this study was that the lowest IL-6 levels were found in normal-weight and overweight individuals, and the lowest CRP levels were found in normal-weight individuals. Such a U-shaped relationship between BMI and proinflammatory factors supports the theory of the “obesity paradox” stating that in the elderly, lower morbidity and mortality are associated with a normal body weight or being overweight [43–46], whereas being underweight or obese (especially morbidly obese) poses a high risk of mortality. Based on the present results, we speculate that this phenomenon might be, at least partially, attributable to an increase in systemic inflammation in extreme weight groups. Notably, in our study participants, a BMI <18.5 kg/m^2^ was associated with the highest IL-6 level and, to a lesser extent, an increase in CRP level. Data that were obtained from the revised Mini Nutritional Assessment Short Form and serum biochemical parameters indicated that these individuals were undernourished and vitamin D-deficient (even though, some of them were still able to meet our phenotypic criteria for successful aging). The available data suggest that poor nutritional status and vitamin D deficiency might be associated with increased levels of proinflammatory factors, such as TNF-α, IL-6, and CRP [47–49]. Therefore, in our underweight elderly patients, high levels of IL-6 and CRP might result from a cumulative action of immunoaging and undernutrition/malnutrition.

We also found that the IL-6 concentration was negatively associated with concentrations of total cholesterol and its high- and low-density fractions in the entire studied population and in its subgroups, while CRP was negatively associated only with HDL. Such findings indicate that low cholesterol levels in the elderly might have adverse effects, such as the increased level of IL-6 that we detected in the present study. Our results are partially consistent with previous studies that reported the existence of similar correlations in oldest-old group of ≥85 year-old individuals [50, 51]. However, we found that such relationships were also true for seniors who were ≥65 years old. Longitudinal studies indicate that aging might be associated with a decrease in total cholesterol and LDL levels and an increase in HDL levels, which is mostly a result of decreasing weight [52]. On the other hand, increased levels of proinflammatory factors are attributable to a higher body weight [53, 54]. Therefore, an inverse relationship between lipids and proinflammatory factors in seniors might be unrelated to total adipose tissue content but may be related to the abdominal localization of adipose tissue [55, 56]. Alternatively, adipose tissue might not be a key factor in the interplay between lipids and inflammation in older age groups.

Consistent with previous findings [23, 57], we found that higher IL-6 and CRP levels were associated with poorer cognitive and/or functional performance, as well as with a higher risk of mortality. This could be explained by the fact that aging-associated chronic inflammation is one of the major causes of aging-related diseases, that might negatively affect the aging phenotype and shorten one’s lifespan [13–22]. However, we found that higher levels of IL-6 and CRP posed an even higher risk of all-cause mortality in physically and cognitively healthy, successfully aging individuals who did not suffer from major aging-related diseases, reflected by 1-year mortality rates (see Figs. 1 and 2 legends), and confirmed by 3-year mortality rates (IL-6: 2.9 % for <1^st^ quartile, 6.9 % for 1^st^-2^nd^ quartile, 14.0 % for 2^nd^-3^rd^ quartile and 25.3 % for >3^rd^ quartile of IL-6 concentration; because there is no established “normal” IL-6 concentration range, we divided the study subjects into IL-6 concentration quartiles; CRP: 7.3 % for ≤3 mg/L, 13.2 % for 3.1-10 mg/L, and 27.2 % for >10 mg/L). Although these deaths could be partially attributed to newly diagnosed aging-associated diseases [21], we speculate that in the individuals who met our criteria of successful aging, elevated levels of proinflammatory factors may serve as an indicator of the increased risk of death or may actually be associated with such risk through the activation of as-yet unidentified pathological mechanisms other than those that lead to cardiovascular disease, diabetes, cancer, or neurodegeneration.

Finally, we found that low-dose acetylsalicylic acid, which is very commonly administered in the elderly population for prevention of cardiovascular events, did not affect IL-6 or CRP levels, which was consistent with recently published results regarding 35 to 75-year-old participants of the Swiss CoLaus Study [58]. This previous study and our present results suggest that the well-studied cardioprotective action of low-dose acetylsalicylic acid may not be attributable to the lowering of IL-6 or CRP levels but rather other mechanisms, such as the modification of other cytokine concentrations.

The strengths of this study reside in the large size of the study group, the mode of recruitment (from urban and rural municipalities), the equally-sized 5-year age cohorts, the equal representation of both sexes, and detailed baseline clinical and biochemical data that allowed us to identify the group of successfully aging individuals based on various parameters [30]. On the other hand, one limitation of the present study was that not all of the study participants had their blood drawn. However, those who did not provide blood were not preselected on the basis of any condition, and we believe that the chance of bias for this reason is relatively low. Another limitation is that the lack of data regarding morbidity after the baseline interview prevented us from determining whether or not and to what extent new cases of aging-related diseases might explain associations between IL-6 and CRP levels and mortality in successfully aging individuals.

## Conclusions

In the present study, IL-6 and CRP levels in the elderly systematically increased in an age-dependent manner in the entire study group, the successfully aging subgroup, and in individuals who suffered from aging-related diseases/disability. Higher IL-6 and CRP levels were associated with poorer cognitive and/or functional performance, and a higher risk of mortality also in successfully aging individuals. Our data support the notion that a single measurement of IL-6 and/or CRP concentrations in elderly individuals is a good predictor of physical and cognitive performance and mortality in the entire elderly population, including successfully aging individuals.

## Abbreviations

ADL, katz activities of daily living scale; BMI, body mass index; CCL2, monocyte chemoattractant protein-1 (chemokine (C-C motif) ligand 2); CRP, C-reactive protein; eGFR, estimated glomerular filtration rate; HDL, high-density cholesterol; HR, hazard ratio; IL-1ß, interleukin-1ß; IL-6, interleukin-6; LDL, low-density cholesterol; MMSE, mini mental state examination; TNF-α, tumor necrosis factor-α.
